# Ectopic positioning of the cell division plane is associated with single amino acid substitutions in the FtsZ-recruiting SsgB in *Streptomyces*

**DOI:** 10.1098/rsob.200409

**Published:** 2021-02-24

**Authors:** Xiansha Xiao, Joost Willemse, Patrick Voskamp, Xinmeng Li, Andrea E. Prota, Meindert Lamers, Navraj Pannu, Jan Pieter Abrahams, Gilles P. van Wezel

**Affiliations:** ^1^ Molecular Biotechnology, Leiden University, PO Box 9505, 2300RA Leiden, The Netherlands; ^2^ Biophysical Structural Chemistry, Leiden University, PO Box 9502, 2300RA Leiden, The Netherlands; ^3^ LIC/Energy and Sustainability, Leiden University, PO Box 9502, 2300RA Leiden, The Netherlands; ^4^ Paul Scherrer Institute, CH-5232 Villigen, Switzerland; ^5^ Leiden University Medical Center, PO Box 9600, 2300RC Leiden, The Netherlands; ^6^ Biozentrum, University of Basel, Mattenstrasse 26, CH-4058 Basel, Switzerland

**Keywords:** cell division, SsgB, mutagenesis, ectopic septa, crystal structure

## Abstract

In most bacteria, cell division begins with the polymerization of the GTPase FtsZ at mid-cell, which recruits the division machinery to initiate cell constriction. In the filamentous bacterium *Streptomyces*, cell division is positively controlled by SsgB, which recruits FtsZ to the future septum sites and promotes Z-ring formation. Here, we show that various amino acid (aa) substitutions in the highly conserved SsgB protein result in ectopically placed septa that sever spores diagonally or along the long axis, perpendicular to the division plane. Fluorescence microscopy revealed that between 3.3% and 9.8% of the spores of strains expressing SsgB E120 variants were severed ectopically. Biochemical analysis of SsgB variant E120G revealed that its interaction with FtsZ had been maintained. The crystal structure of *Streptomyces coelicolor* SsgB was resolved and the key residues were mapped on the structure. Notably, residue substitutions (V115G, G118V, E120G) that are associated with septum misplacement localize in the *α*2–*α*3 loop region that links the final helix and the rest of the protein. Structural analyses and molecular simulation revealed that these residues are essential for maintaining the proper angle of helix *α*3. Our data suggest that besides altering FtsZ, aa substitutions in the FtsZ-recruiting protein SsgB also lead to diagonally or longitudinally divided cells in *Streptomyces*.

## Graphical abstract

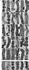

## 1. Introduction

During bacterial cell division, the tubulin homologue FtsZ is the first component recruited to the division site, where it polymerizes into a ring-like structure known as the Z-ring, which contracts during cytokinesis [1]. FtsZ is a self-activating GTPase that undergoes guanosine-5-triphosphate (GTP)-dependent assembly into protofilaments by the head-to-tail association of individual monomers [2–4]. Super-resolution microscopy revealed that the Z-ring is discontinuous and consists of FtsZ assemblies [5,6]. Recent studies suggested that FtsZ filaments undergo treadmilling circumferentially around the division plane and drive the movement of the peptidoglycan (PG) -synthesizing machinery [7–9]. New cell wall components are inserted progressively at the septum, ultimately resulting in cell constriction. The treadmilling is associated with the conformational change and the kinetic polarity of the FtsZ filament and controls the rate of PG synthesis and cell division [8,10]. Septum-site localization in unicellular bacteria depends on FtsA and ZipA, which anchor FtsZ polymers to the cell membrane, with ZapA stabilizing the FtsZ filaments and promoting lateral interactions [11–13]. In *Escherichia coli*, control of Z-ring timing and localization is governed by the Min system and by nucleoid occlusion, which negatively regulate FtsZ polymerization [14–17].

Owing to its central role in cell division, FtsZ is essential in nearly all bacteria. Two exceptions are the parasite *Mycoplasma* [18], which has a reduced genome size and no cell walls, and *Streptomyces* [19,20]. Streptomycetes are filamentous Gram-positive bacteria in the phylum of Actinobacteria that have a complex mycelial lifestyle [21]. These bacteria produce over 60% of all known antibiotics and many other bioactive natural products [22,23]. Streptomycetes are model organisms for the study of multicellularity and bacterial morphogenesis [24,25]. Exponential growth of the multi-nucleoid vegetative hyphae is achieved by apical growth and branching. At this stage of the life cycle, cell division does not affect physical separation of the cells, but instead long syncytial cells are formed that are separated by cross-walls [26]. When the developmental programme is switched on, streptomycetes produce aerial hyphae that ultimately differentiate into chains of unigenomic spores. Recent studies highlight that local membrane synthesis and branching may be an important divisome-independent mechanism for cell proliferation in *Streptomyces* [27,28].

Streptomycetes lack the canonical cell-division regulation systems such as Min and Noc [29]. Instead, a positive control system has evolved, whereby FtsZ is actively recruited to the septum sites by SsgB, in concert with its paralogue SsgA [30]. The onset of sporulation and the correct localization of SsgB to the future septum sites are regulated by a transmembrane protein SepG [31]. In addition, two dynamin-like proteins and SepF are involved in stabilization of the Z-ring during *Streptomyces* sporulation [32], where the assembly of Z-ring is influenced by a small protein called CrgA [33]. The SsgA-like proteins (SALPs) are a family of regulatory proteins that is unique in sporulating actinobacteria [34,35]. SsgB is the archetypical SALP, with a conserved function in the development of actinomycetes [36]. While the SsgB protein sequence varies strongly between less related Actinobacteria, the protein is extremely well conserved within a genus, with a maximum of one amino acid (aa) variation, a feature that has been applied for the phylogenetic analysis of closely related Actinobacteria [37]. Actinobacteria that form single spores, such as *Micromonospora* or *Thermobifida*, only have one SALP, namely the FtsZ-recruiting protein SsgB, while up to 14 SALPs can be found in those genera that form chains of spores, such as *Streptomyces* [38]. The crystal structure of a SsgB orthologue from *Thermobifida fusca* was resolved, which revealed a ‘bell-shape’ trimer that is assembled mainly through *α*-helix interactions.

In this work, we report on the importance of individual residues in the localization of the septum during sporulation-specific division, by creating a library of SsgB mutants and studying their effect on cell division and morphogenesis. Single aa changes in SsgB had major effects on cell division, spore-wall synthesis and DNA condensation and/or segregation. Remarkably, specific mutations led to the formation of additional septa with 10° to 90° rotation of the division plane. Altered division planes have so far only been observed in mutants of FtsZ itself [39–41], and in worm-associated bacteria *Candidatus *Thiosymbion oneisti** and *Thiosymbion hypermnestrae*. In the latter two bacteria, cell growth and longitudinal division are polarized by their symbiotic nematode hosts [42]. X-ray crystallography revealed that SsgB from *Streptomyces coelicolor* resembles its distant orthologue from *T. fusca* (PDB ID: 3CM1). Key mutational residues were mapped onto the structure of SsgB. Further structural analyses and molecular simulation were conducted that highlight the important role of *α*2–*α*3 loop region to the function of SsgB. Our data support the predominant role of SsgB in the accurate positioning of the division site and the placement of the Z-ring.

## 2. Results

### 2.1. Mutational analysis of *ssgB*

SsgB shows unusual conservation, with near complete conservation within a genus and high divergence even between related actinobacterial genera (electronic supplementary material, figure S1). To investigate this further, we analysed the effect of amino acid substitutions in *ssgB* on cell division and morphogenesis, using *S. coelicolor* as the model system. For this, we first created a random mutant library using error-prone PCR, similar to the approach used previously for the mutational analysis of SsgA [43]. Mutant genes, as determined by sequencing, preceded by (and transcribed from) the original *ssgB* promoter region, were cloned into the low-copy number vector pHJL401 and introduced into the *ssgB* null mutant, followed by scrutiny of sporulation and cell division. To ascertain that the observed phenotypes were not owing to differences in SsgB expression, the mutant was also complemented with pHJL401 expressing wild-type SsgB from its original promoter, which gave a full wild-type sporulation phenotype. To exclude plasmid loss, plasmids from transformants expressing SsgB variants were isolated and sequenced before further analysis. Additionally, Western analysis was performed using anti-SsgB antibodies (electronic supplementary material, figure S2). Samples were equalized for protein load and normalized based on the levels of elongation factor EF-Tu1 [44]. This revealed an average expression level of 77 ± 10% of the wild-type level.

Spores of *S. coelicolor* are grey-pigmented owing to the production of the WhiE spore pigment [45], while colonies developing non-sporogenic aerial hyphae are white; intermediate phenotypes (reduced sporulation results in a light-grey pigmentation) also occur. This feature was used to subcategorize all transformants into three groups: white, light grey and grey. The mean grey level of growing patches was analysed based on the scanner images. By this approach, the degree of sporulation could be readily monitored (electronic supplementary material, figure S3 and table S1). 232 clones were isolated from the transformants and sequenced. Of these, 84 had no or silent mutations, 39 had multiple mutations and 65 had insertions or deletions. Of the 42 remaining clones, 35 unique single substitutions were identified and these were analysed further. Out of 35 SsgB variants, six failed to sporulate and the others showed significant sporulation defects or reduced sporulation (electronic supplementary material, figure S4 and table S1).

**Figure 1.**
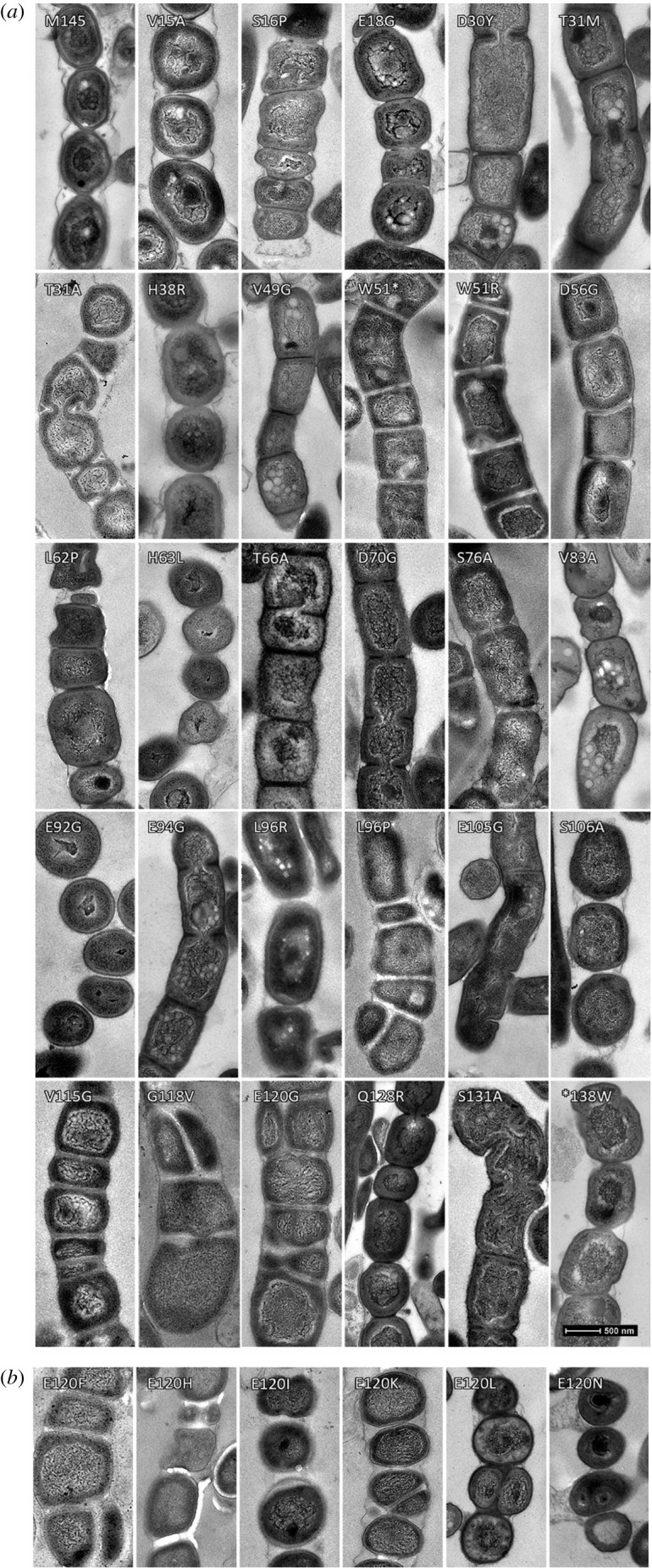
Transmission electron micrographs of sporogenic hyphae from the wild-type and single sporulating SsgB mutants. (*a*) D30Y, V49G, T66A, D70G, E94G and S131A result in thinner cell walls. L96P, V115G, G118V and E120G give rise to septum rotation, which also affect DNA condensation. Disturbed DNA segregation was observed in L88R (see electronic supplementary material, figure S4). Mutants expressing SsgB variants V15A, S16P, E18G, T31A, T31M, H38R, W51R, L62P, H63L, S76A, V83A, E92G, L96R, E105G, S106A and Q128R showed aberrant spore sizes (see also electronic supplementary material, table S2). (*b*) Six additional amino acid substitutions of E120 that lead to longitudinal division. (E120G is shown in figure 1*a*). Bars: 500 nm for TEM micrographs.

To obtain more detailed insights into the morphological changes correlating to the substitution mutants, the transformants expressing SsgB variants were subjected to transmission electron microscopy (TEM) (figure 1). Wild-type spores were homogeneous in size, with a thick electron-dense spore wall and condensed DNA in the centre of the spores. Conversely, spores from transformants expressing SsgB variants generally showed high variation in spore-wall thickness, spore size and shape, and frequently also aberrant DNA segregation and/or condensation (figure 1 and electronic supplementary material, table S2). Much to our surprise, in some cases up to 90° rotation of the septal plane was seen, dividing the spores parallel to the growth direction of the hyphae. This suggests that mutation of single SsgB residues may affect the coordination of cell division in aerial hyphae of *Streptomyces*, as detailed below.

### 2.2. Rotation of the division plane owing to single amino acid substitutions in SsgB

Based on the outcome of the random mutant library, 22 residues were selected for site-saturated mutagenesis, namely W51, L88, A95, L96, L97 and the C-terminal residues 115–131 that are centred around E120 that correlated to the surprising longitudinal division (electronic supplementary material, table S3). Each of these residues was changed into on average 14 different aa residues using DNA synthesis. Mutants for the hydrophobic residues W51, L88, A95, L97, V115, P116 and P117 frequently had non-sporulating phenotypes (electronic supplementary material, table S3). Aberrant spore sizes were seen in most of the mutants, with some also showing irregular cell wall thickening (i.e. D70G; figure 1) and abnormal DNA segregation (L88R) (electronic supplementary material, figure S5 and table S2). Importantly, seven mutants wherein E120 was replaced by F, G, H, I, K, L or N produced septa with significant rotation of the division plane—in addition to canonical septa. The angles of these ectopically positioned septa ranged from diagonal to longitudinal (i.e. 90° rotation, with septa parallel to the hyphal wall), of which 22.7% were positioned diagonally (336 rotated septa out of 1479, 22.7%). Out of 336 rotated septa, 7.3% were longitudinally positioned. See figure 1*b*; electronic supplementary material, figure S6. In addition to mutants expressing SsgB E120 variants, longitudinal division was also seen in mutants expressing SsgB variants V115G, G118V, L96I, L96P and L96S, whereby the latter three produced relatively few ectopic septa, with an average of 1.7% rotated septation. To ascertain that longitudinal division does not occur in the wild-type strain under the chosen conditions, over 1011 spores of the wild-type strain were checked by scanning electron microscopy (SEM) and TEM, and not a single rotated septum was observed.

**Figure 2.**
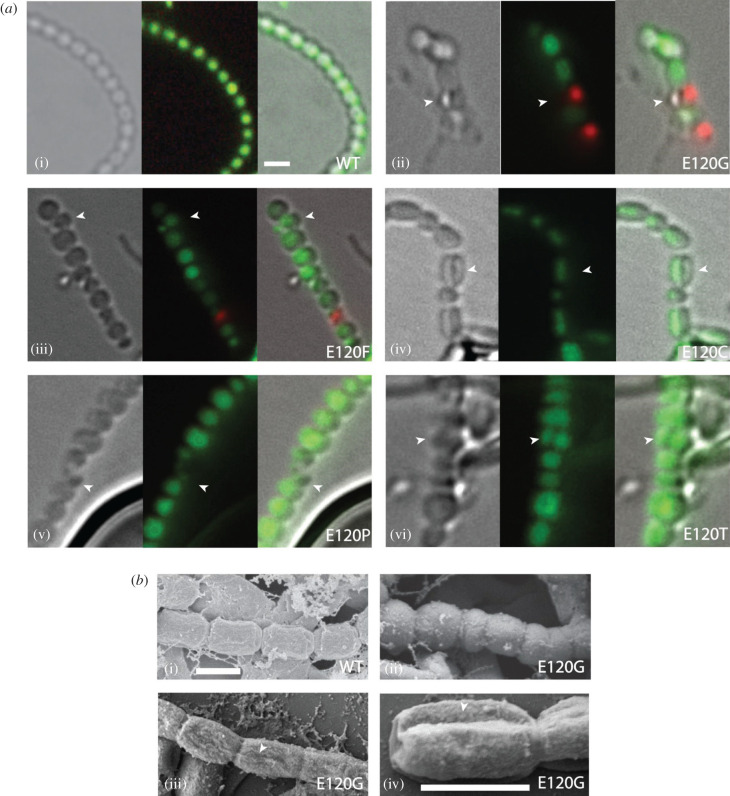
Impression prints of spores from cells expressing wild-type SsgB or SsgB E120 mutants. (*a*) Longitudinal cell division (pointed by arrowhead) that are shown by fluorescent microscope from different E120 mutants. *Left*, bright-field images; *middle*, Syto9/PI stained images; *right*, overlays of the two images. All the spores were obtained after 7 days of growth from wild-type cells or from transformants of its *ssgB* null mutant expressing SsgB E120 mutants. Bars, 1 µm. (*b*) SEM imaging of wild-type SsgB and its E120G mutant. Panel (ii) shows the variability in spore size, whereas panels (iii) and (iv) show the start of longitudinal indentations and a later stage of longitudinal division, respectively.

In order to see if the longitudinal septation also resulted in physical separation of the severed spores, we made impression prints of the strain expressing SsgB variant E120G, which had been grown for 7 days on soya flour mannitol (SFM) agar plates. These spores were then fixed with 1.5% glutaraldehyde in PBS, followed by dehydration using a graded series of acetone (70–100%). This experiment clearly demonstrated that the strain expressing SsgB-E120G produces spores that are longitudinally sectioned in two and that this process is completed by spore fission (figure 2*b*, panels (iii–iv)). Panel (ii) shows the size variability of SsgB-E120G mutant spores, and panels (iii) and (iv) present evidence of longitudinal indentations and a later stage of longitudinal division.

Viability of the spores of mutants in which the residue E120 had been replaced by other aa was compared to those of wild-type spores. For this, impression prints were stained with Syto9 for viable spores and propidium iodide (PI) for dead spore, and imaged via fluorescence microscopy (FM). While wild-type SsgB spores were nearly all viable, those obtained from E120 substitution mutants varied a lot, with 5–70% dead spores, depending on the mutant (electronic supplementary material, figure S7). Like in the SEM experiments, longitudinal septation could also be seen from the outside using light microscopy, indicative of unique cell fission parallel to the hyphal wall (figure 2*a*, panels (ii–vi)). Some empty spore compartments were seen in the live/dead staining in the longitudinally septated spores (panels (ii) and (iv)), which can be explained by the unequal DNA septation during cell division in *ssgB* mutant. This resembles the minicompartments that were seen in *parA* deletion mutant, where the prespores are anucleate [46]. The rate of the physical separation of the severed spores was counted for E120 substitution mutant, which revealed a range of 3.3–9.8% split spores as compared to the normal separation spores (electronic supplementary material, table S4).

### 2.3. Localization and dynamics of SsgB variants

To test whether longitudinal division in the aerial hyphae correlated to the localization of SsgB, chimaeric SsgB-eGFP, SsgB-E120G-eGFP and SsgB-G118V-eGFP fusions were created as described [30]. While wild-type SsgB showed the typical pattern of foci on either side of the hyphal wall (electronic supplementary material, figure S8, panel a), the G118V variants localized more centrally and also longitudinally (electronic supplementary material, figure S8, panels b). This resulted in both canonical septal rings (perpendicular to the hyphal wall) and with a certain frequency also septa that were tilted by 90^o^ (marked by arrowheads in electronic supplementary material, figure S8, panel b).

**Figure 3.**
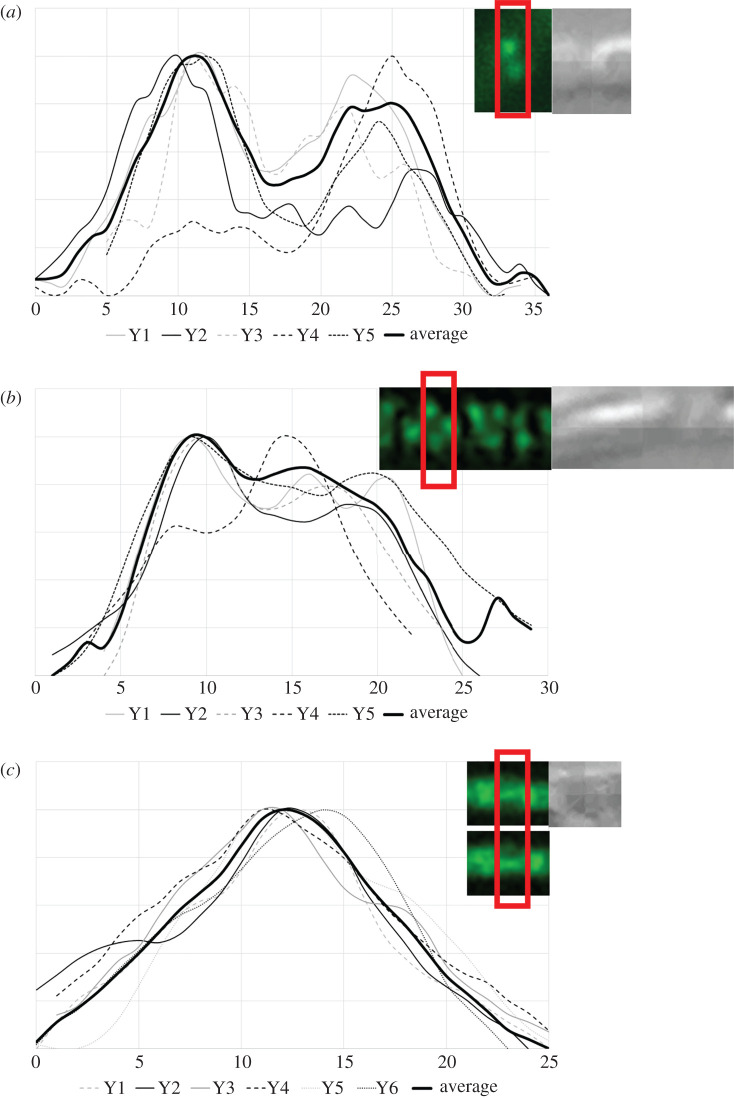
Intensity plots of SsgB foci on the septa. (*a*) The wild-type SsgB-localization during early cell division shows two foci on either side of the hyphal wall; (*b*) Mutants expressing SsgB (E120G) showed aberrant localization, whereby SsgB was located all over the hyphal wall; (*c*) Occasional longitudinal septation was seen in SsgB (G118V), whereby eGFP fusions of SsgB mutant proteins localized parallel to the hyphal wall and in the middle of the hyphae. The red box indicates the width of the box that was used for making the profiles. Y1–Y5, single intensity plots of selected foci; *X*-axis, the distance between two foci on either side of hyphal wall; *Y*-axis, the fluorescent intensity.

Fluorescence intensity distribution of SsgB-E120G-eGFP and SsgB-G118V-eGFP was compared with SsgB-eGFP. For comparison the same width of the hyphae was selected and five different septa were measured (Y1–5, figure 3). The plotted graph of wild-type SsgB indicates its localization on either side of the hyphae wall. Variants of SsgB-E120G and SsgB-G118V showed aberrant localization, which occasionally resulted in longitudinal septation; this correlated to localization of SsgB in the middle of the hyphae and perpendicular to the canonical septal plane. To gain insights into the dynamic association/dissociation of SsgB with the divisome, monomeric exchange was examined via fluorescence recovery after photobleaching (FRAP) for the E120G and G118V mutant. The recovery time after photobleaching was determined both on pre-septation foci as well as on septa. No difference in dynamics was seen between wild-type SsgB and its G118V variant. Both showed a recovery time of around 60 s (electronic supplementary material, figure S9), while the E120 variant differed slightly from the wild-type SsgB, with a recovery time of around 42 s, which is similar to previously reported data [30].

**Figure 4.**
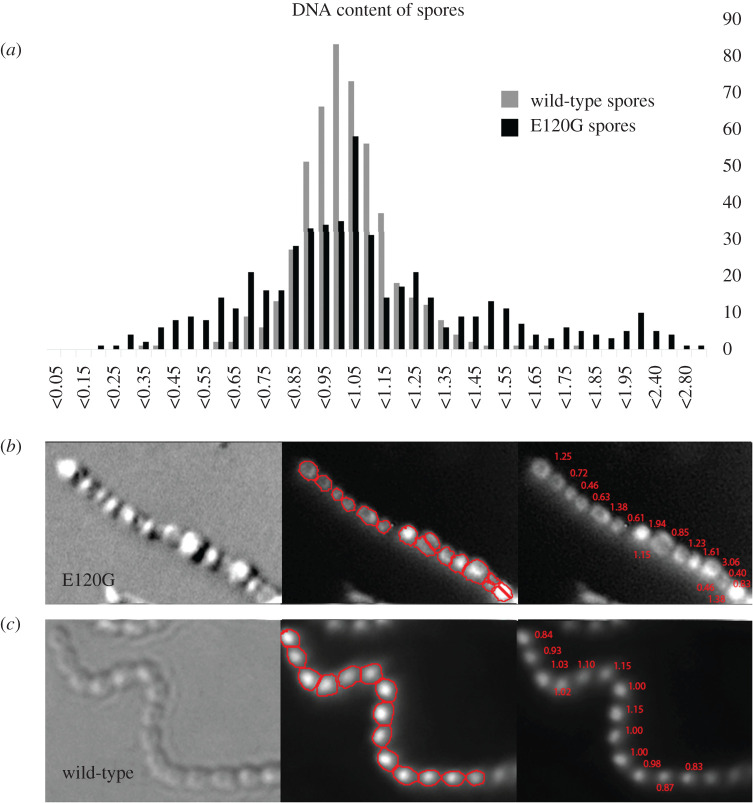
DNA content analysis of SsgB variants. (*a*) The DNA content distribution in both wild-type (light grey) and E120G spores (black) is shown. The median DNA content of the spores was set to 1 to normalize the data. A normal distribution was observed for wild-type strain, whereas the strain expressing SsgB (E120G) had much more variation in DNA content. *Y*-axis, the frequency at which each DNA content was observed; *X*-axis, the DNA content in each spore. (*b*) To illustrate, one spore chain containing longitudinal divisions is shown with the respective DNA content in each spore (between 0.4 and 3.0 chromosomes). (*c*) In comparison, wild-type spores showed relatively little variation (between 0.83 and 1.15 in the spore chain).

The relative DNA content per spore of over 500 spores of wild-type strain and SsgB-E120G mutant was studied. While wild-type spores showed a normal DNA distribution, SsgB-E120G mutant showed major variation in the relative DNA content per spore (figure 4). As an illustration, one spore chain containing longitudinal divisions is shown with the respective DNA content in each spore, which revealed 0.4–3.0 chromosomes for each spore in SsgB-E120G mutant (figure 4*b*). Conversely, 0.83–1.15 chromosomes were observed in the wild-type strains for each spore (figure 4*c*).

To establish whether SsgB-E120G had retained the ability to interact with FtsZ, wild-type SsgB, SsgB variants E120G and E120A, as well as a C-terminally truncated version of SsgB (SsgBΔC, spanning 1–114 aa) were expressed and purified and then tested using a pelleting assay. FtsZ polymers formed in the presence of GTP. When wild-type SsgB or its E120A, E120G and SsgBΔC variants were added to the reaction mixture, both FtsZ and all SsgB variants were enriched in the pellet fractions, whereby the amount of FtsZ that pelleted was the same in all fractions, with or without SsgB (electronic supplementary material, figure S10A). The pelleting assay provides strong evidence that all of the SsgB variants can interact with FtsZ. We then tested the polymerization of FtsZ by the same SsgB variants using negative staining. Bundles were formed when wild-type SsgB or SsgB-E120G was mixed with FtsZ (electronic supplementary material, figure S10B). However, in the presence of SsgB E120A and SsgBΔC, less extensive bundling was observed. In addition, bacterial two-hybrid studies (BACTH) revealed that the interaction of SsgB with itself was not hampered by the E120G substitution, while the interaction with SsgA was reduced (electronic supplementary material, figure S11). BACTH of wild-type SsgB or SsgA with SsgB variants V115G, G118V and L96P were also measured. While SsgB V115G and G118V showed similar interaction with wild-type SsgB and its E120G variant, the interaction of SsgB L96P with either SsgB or SsgA was largely reduced.

### 2.4. Crystal structure of *Streptomyces coelicolor* SsgB

**Figure 5.**
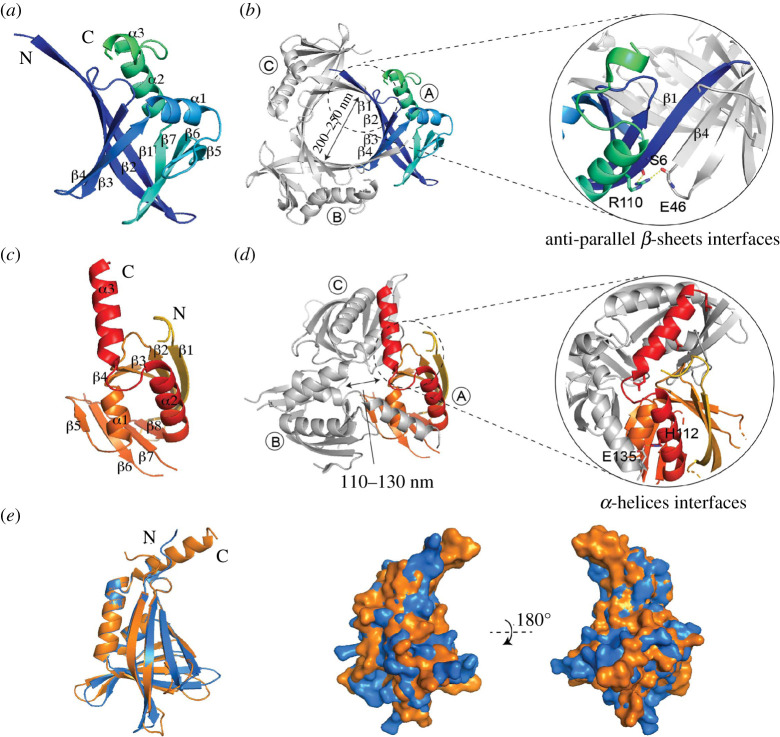
Crystal structure of the *Sc*SsgB trimer. (*a*) Ribbon diagrams showing the monomer structure of SsgB from *S. coelicolor*. (*b*) The overall structure of *Sc*SsgB reveals a trimer. Structure statistics are listed in electronic supplementary material, table S6. The interface between adjacent monomers is formed by two antiparallel *β*-sheets. (*c*) The monomer structure of SsgB from *T. fusca* (PDB code 3CM1)*.* (*d*) The interface between adjacent monomers of *Tf* SsgB is formed by *α*-helices. (*e*) Overlap of *Sc*SsgB (blue) and *Tf* SsgB (orange) subunits. *Left*, side view of the electrostatic surface alignment of *Sc*SsgB and *Tf* SsgB structure. *Right,* the same electrostatic figure but rotated by 180°.

In order to gain more insights into the structure–function relationship for key SsgB residues, the structure of *S. coelicolor* SsgB (*Sc*SsgB) was resolved via X-ray crystallography. For this, tetragonal crystals of *Sc*SsgB were obtained, and based on this, a homo-trimer was resolved at 2.1 Å (PDB ID Code 6SLC) with eight molecules per asymmetric unit (electronic supplementary material, table S6). The 13 aa residues at the C-terminus were highly mobile and could therefore not be modelled, owing to lack of electron density. Each subunit was arranged as an α + β fold, with seven *β*-strands packed into a barrel structure, covered by three *α*-helices (figure 5*a,b*), which strongly resemble those of *T. fusca* SsgB (*Tf* SsgB) structure [36] (PDB ID: 3CM1, figure 5*c*,*d*). The root-mean-square deviation (r.m.s.d.) is 1.9 Å with 92% of all residues aligned in *Sc*SsgB and 87% in *Tf* SsgB (aa sequence identity between these two proteins is 46%). A superimposition of *Sc*SsgB and *Tf* SsgB subunits is shown in figure 5*e*. *Sc*SsgB trimer adopts a ‘whirly’ shape and is assembled through an antiparallel *β*-sheet interaction between *β*1 from one subunit and *β*4 from the neighbouring subunit (figure 5*b*; electronic supplementary material, figure S12). By contrast, the *Tf* SsgB trimer is assembled mainly through *α*-helices (figure 5*d*). The *Sc*SsgB trimer forms a 12-stranded beta-barrel with an inner diameter of about 20–25 Å (figure 5*b*).

Crystallographic data obtained for SsgB from *T. fusca* [36] and for *S. coelicolor* (this work) suggested that SsgB forms trimers. To ascertain this, size-exclusion chromatography (SEC) of wild-type *Sc*SsgB and *Sc*SsgBΔC was conducted. SEC results revealed that purified *Sc*SsgB and *Sc*SsgBΔC mainly existed as a monomer in solution (electronic supplementary material, figure S13), which is consistent with our previous multimerization studies of *T. fusca* SsgB [36], indicating that a possible equilibrium exists between the monomeric and trimeric states.

### 2.5. Analysis of single aa substitutions and mapping of key residues

**Figure 6.**
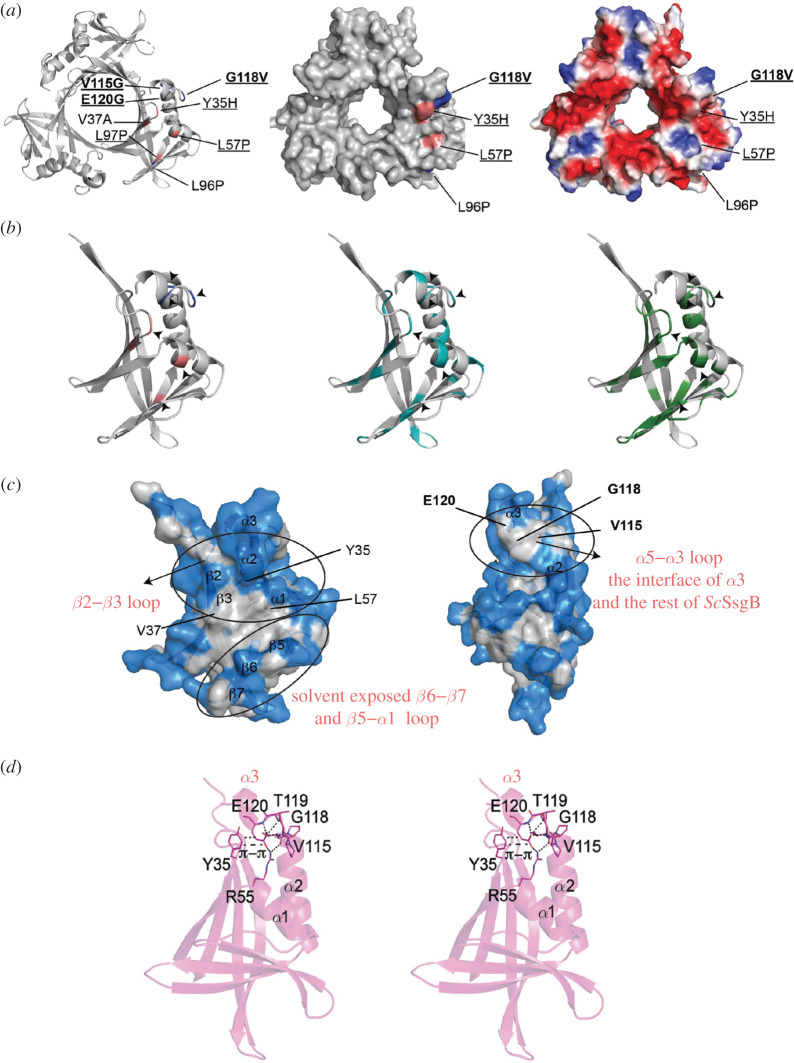
Key mutations and their interactions in SsgB trimer structure. (*a*) *Left*, aa substitutions L96P, V115G, G118V and E120G show tilted division; aa substitutions of Y35H, V37A, L57P and L97P (deep salmon) result in non-sporulating phenotype; *middle*, *right,* top view of all the functional aa substitutions mapped on the surface structures. Conserved residues are underlined. (*b*) *Left*, key residues as shown by (*a*); *middle*, residues corresponding to all substitutions in this study (cyan); *right*, conserved residues (green). (*c*) Key residues (Y35H, V37A, L57P and L97P) are located in the hydrophobic patch, while V115, G118 and E120 are clustered on the *α*2–*α*3 loop region that mediates the interactions of *α*3 and the globular domain of SsgB. White patches in the surface structure indicate hydrophobic residues. (*d*) Stereo-view of E120 in the monomer structure and its interactions with the surrounding residues.

Key residues that correlated to the occurrence of longitudinal cell division were mapped onto the *Sc*SsgB trimer structure (figure 6*a*; electronic supplementary material, figure S14A). Mutations that correlate to residues that are evolutionarily conserved in all SALPs are underlined (figure 6*a*; electronic supplementary material, figure S14B). Residues V115, G118 and E120 cluster together, and are centered on the lid of the beta-barrel consisting of *α*1, *α*2–*α*3 and *β*1–*β*2 loop, which is close to the interface between *α*3 and the rest of the subunit (figure 6*a*,*b*). Consistent with their strategic localization in the *α*2–*α*3 loop (figure 6*c*), aa substitutions V115G, G118V or E120G resulted in the formation of aberrant tilted septa in addition to the canonical septa perpendicular to the hyphal wall, with some septa showing full 90° rotation of the division plane. Residue E120 plays a key role in maintaining the proper angle between *α*3 and the rest of the protein. Three hydrogen bonds are formed between the E120 side chain and the main chains of E120, T119 and G118 in the *α*2–*α*3 loop region (figure 6*d*). Besides, E120 and V115 provide two additional salt bridges to R55. Interestingly, a π–π interaction and a hydrogen bond were observed for E120-Y35 and H121-Y35. All these interactions stabilize the angle of *α*3, supporting the importance of the proper angle between *α*3 and the rest of the protein to the function of *Sc*SsgB.

### 2.6. Molecular dynamic simulation of SsgB-E120G

**Figure 7.**
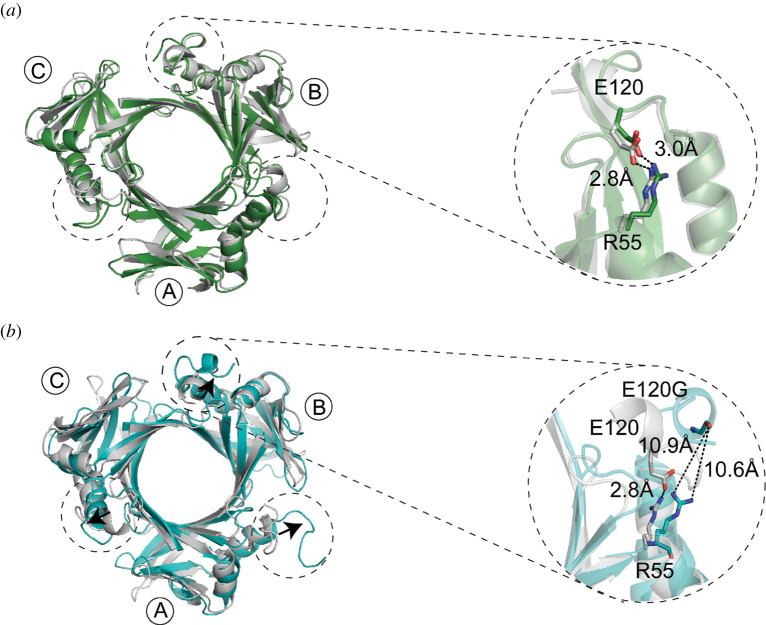
Molecular dynamic simulation of SsgB wild-type and E120G mutant. (*a*) MD result of SsgB wild-type structure. The *α*3 helix stays at the same orientation and the distance between R55 and E120 keeps at 2.8 Å and 3.0 Å, before (green) and after MD (grey). (*b*) The arrows indicate the changed angle of SsgB-E120G mutant (cyan) compared to the wild-type SsgB (grey) after MD.

Our work demonstrated that residue E120 plays a key role in maintaining the proper angle of *α*3 relative to the rest of the protein. Mutation of this residue would disrupt the interaction, and changing the angle of *α*3 may drive rotation of the septum plane, and thus explain the observed longitudinal cell division. The heading and the high B-factor of the existing residues in the *α*3-helix (electronic supplementary material, figure S15) suggest that it can extend flexibly to the centre of the trimer. Despite many attempts under different crystallization conditions, we failed to obtain crystals for SsgB-E120G or SsgB E120A*. In silico* molecular dynamics revealed that while the *α*3 helix of the wild-type SsgB protein is not affected significantly by the simulations, with a distance of 2.7 and 3.0 Å, respectively, between E120 and R55 (figure 7*a*), the *α*3 helix of SsgB-E120G flips some 90° outwards of the tight trimer, with the distance increasing to 10.6 and 10.9 Å, respectively (figure 7*b*). This provides supportive evidence that the angle of the *α*3 helix plays a key role in the proper functionality of SsgB.

## 3. Discussion

SALPs play a central role in controlling the steps of sporulation-specific cell division in *Streptomyces*. Inspired by the extremely high conservation of the SsgB protein in *Streptomyces* species, with natural variants only found in aa 128 (Q, R or T), we studied the effect of point mutations in the protein on cell division and morphogenesis of the model strain *S. coelicolor*. As expected, many mutants showed morphological defects relating to cell division and sporulation, including varying spore sizes, aberrant DNA segregation and condensation, and cell wall thickening. However, SsgB substitutions L96P, V115G, G118V and various aa substitutions in residue E120 caused rotated to longitudinal cell division in addition to the canonical perpendicular septa. As a result, spores were formed that were diagonally or longitudinally sectioned, visualized both inside spores (via TEM and FM) and outside (via SEM and FM). These results highlight the dominant role of SsgB in the control of cell division and Z-ring formation in *Streptomyces*, which is also consistent with the observation of the extremely high level of conservation of SsgB within *Streptomyces* species.

It is important to note that diagonal or longitudinal septa always coincided with canonically oriented septa as seen by TEM and FM. Furthermore, the longitudinal septa always connected two canonical septa: we never observed these additional Z-rings formed between septa and the hyphal wall. And finally, the longitudinal septa could be formed with different spacing relative to the hyphal wall, allowing asymmetric cleavage of spores. This suggests that during normal septation, a second septum is formed that grows sideways, under different angles. Evidence of longitudinal septation was also frequently seen as diagonally or longitudinally severed spores. This shows that these ectopic cell division events were completed via cytokinesis. However, the mechanism of longitudinal cell division is unclear and needs further analysis; we cannot exclude that rotated septa formed in prespore compartments could be essentially spherical or cylindrical. DNA content and viability studies of SsgB E120 substitution mutants revealed abnormal chromosome distribution (0.4–3.0) and relatively reduced viability (5–70% dead spores) compared to the wild-type strain. Besides affecting septum positioning, mutations of SsgB also resulted in defects in spore viability, spore shape, wall thickness and DNA condensation and/or segregation. Analysis of substitutions of residue L96 revealed that exactly two thirds of all spores had disturbed DNA condensation. Aberrant DNA segregation was also seen frequently in SsgB L88R mutants. These data indicate that SsgB may play key roles beyond septum positioning. BACTH studies revealed that the interactions of SsgB variants V115G, G118V and E120G with wild-type SsgB have been retained, while the interactions of these variants with SsgA were largely reduced. Notably, for SsgB variant L96P, its interactions with SsgB and SsgA were all blocked. SALPs such as SsgA–G play important and highly specific roles in the control of the sporulation process [34]. Previous studies showed that mutants lacking *ssgC* are disturbed in DNA segregation/condensation, those lacking *ssgD* are defective in spore-wall synthesis, those lacking either *ssgE* or *ssgF* have defects in autolytic spore separation and *ssgG* is required for correct septum localization [34]. It is very possible that altered interaction between SsgB and one or more other SALPs, or perhaps other components of the divisome, may contribute to the ectopic septum placement. This requires further verification.

SsgB-E120G, E120A and SsgB*Δ*C had retained the ability to interact with FtsZ *in vitro*. However, instead of promoting the filamentation of FtsZ, SsgB and its variants may assist in the bundling of FtsZ filaments as observed by negative staining. *In vivo* localization studies showed that the foci of SsgB-E120G-eGFP and G118V-eGFP simultaneously formed both canonical and longitudinal septa. FRAP studies on SsgB-E120G-eGFP and G118V-eGFP further confirmed the *in vivo* studies by showing that these variants had the same dynamics as wild-type SsgB; this was true for foci that were localized canonically (adjacent to the cell wall) and for those that were centrally located.

Notably, protein structural analyses revealed that residues that are associated with septum misplacement are located in the *α*2–*α*3 loop region that link the *α*3 helix and the rest of the protein. Several interactions, including hydrogen bonds (E120–E120, E120–T119 and E120–G118, H121–Y35), salt bridges (E120–R55 and V115–R55) and π–π interaction (E120–Y35) likely stabilize and maintain the proper angle of the *α*3 helix with respect to the rest of the protein. Mutating E120 likely disrupts this critical interaction, and thus results in major changes in the orientation of *α*3, by up to 90°. Mutations of the interacting partners all showed functional defects, as seen from the blocked cell division (Y35H), septum rotation (V115G, G118V), DNA segregation (V115G, G118V) and heterogeneity in spore sizes (V115G, G118A, H121I). Moreover, some longitudinal septa were seen in L96P mutants, which again can be explained by changes in the orientation of helix *α*3, as the mutation will disrupt the interaction between the neighbouring *α*2 helix and *β* 7-strand. Molecular dynamic (MD) simulation further confirmed that the E120 substitutions are correlated to the motion of *α*3 helix of SsgB. These data support the significance of the orientation of helix *α*3 to the function of SsgB. However, whether changing the position of helix *α*3 is the direct factor that causes the observed diagonal and longitudinal cell division events remains to be established.

Naturally occurring longitudinal fission had been reported in the insect endosymbiont *Spiroplasma poulsonii* and two marine nematode-attached Gammaproteobacteria, *Candidatus Thiosymbion oneisti* and *Thiosymbion hypermnestrae* [42,47]. In *Spiroplasma*, cells are divided in a Y-shape mode that links to Z-ring reorientation, while in the Gammaproteobacteria, the machineries for growth and division are not reoriented; instead, they mesh to a point at which they are considered as ‘squeezed’ *E. coli* cells. Mutants in FtsZ that alter the division plane have also been reported in *E. coli, B. subtilis* and *Streptomyces* spp. expressing mutant FtsZ species [39–41]. In temperature-sensitive *ftsz26* (Ts) mutants of *E. coli*, FtsZ forms spirals that further contract and form spirally invaginating septa [40]. The abnormal septal invaginations were also observed in a similar *ftsZ (ts1)* mutant in *B. subtilis* [41]. Specific aa substitutions F37I and F37R of FtsZ led to spiraling septation in *Streptomyces *spp*.* [39]. These results suggest that the geometry of division could occur in the perpendicular plane as seen in *Staphylococcus aureus* [48]. Our work shows that rather than aa substitutions in FtsZ itself, changes in FtsZ-recruiting proteins such as SsgB in *Streptomyces* also lead to diagonally or longitudinally divided spores. Further studies to localize FtsZ in SsgB-G118V, E120G and E120A mutants are underway to understand the mechanism behind longitudinal cell division in *Streptomyces*. It will also be important to study the interactions between SsgB-G118V, E120G and E120A proteins and other cell division components and to determine whether mutations in the *α*2–*α*3 loop affect interactions with other proteins.

## 4. Conclusion

Our work shows that single aa substitutions in SsgB affect cell wall synthesis, septum orientation and DNA condensation and segregation during sporulation of *S. coelicolor*, including unique longitudinal division events that have not been seen previously and lead to horizontally severed spores. The crystal structure of *S. coelicolor* SsgB was resolved and key mutational residues were mapped onto the structure. Notably, residues that are associated with septum misplacement are located in *α*2–*α*3 loop region that links the *α*3 helix and the rest of the protein. Further protein structural analyses and MD simulation showed that residues V115, G118 and E120 are essential to maintain the proper angle of the *α*3 helix, whereby substitution of aa V115G, G118V or E120G ultimately leads to cell fission along the long axis of the spores. Our work provides important new structural and biochemical insights into the function of the key cell division control protein SsgB during sporulation-specific cell division in *Streptomyces*.

## 5. Material and methods

### 5.1. Strains and culturing conditions

All strains described in the paper are listed in electronic supplementary material, table S7. *S. coelicolor* M145 was obtained from the John Innes centre strain collection. Its *ssgB* null mutant was described previously [49]. Transformants harbouring pHJL401 plasmids were grown on SFM agar plates containing apramycin (for the *ssgB* deletion) and thiostrepton (to maintain the plasmid) to final concentrations of 50 µg ml^−1^ and 25 µg ml^−1^ at 30°C. For growth in liquid medium, the recombinants were grown in a 1 : 1 mix of TSBS and YEME at 30°C. *Escherichia coli* JM109 was used for amplification of plasmids. *Escherichia coli* Rosetta™ 2 (DE3) pLysS strain was used for overexpression of all the proteins used in this paper. *Escherichia coli* BTH101 was used for BACTH assay. Protoplasts used for transformation were prepared based on previously described protocols [50,51] (see electronic supplementary material, method).

### 5.2. Random mutagenic PCR

The SsgB promoter region and coding sequence were amplified separately from the *S. coelicolor* chromosome using oligonucleotide pairs pSsgB_fw + pSsgB_rv and SsgB_fw + SsgB_rv, respectively (electronic supplementary material, table S8). An NdeI restriction site was introduced overlapping the translational start codon to enable ligation of these fragments after mutagenic PCR. Because pUC19 and pHJL401 already contain an NdeI site, these were removed prior to construct creation using the DNA polymerase I Klenow fragment method. The * behind the plasmid indicates that an NdeI site was removed. The *ssgB* gene (EcoRI-HindIII) was cloned into a variant of pUC19*, thereby creating pJPM1. Construct pJPM2 was based on the low-copy number shuttle vector pHJL401* and contained the PCR-amplified *ssgB* promoter fragment (cloned as EcoRI-HindIII fragment). Mutations in *S. coelicolor ssgB* were introduced by random mutagenic PCR using pJPM1 as template, as described [43]. The desired mutation frequency of 1–3 mutations was checked both on gel and by sequencing. The 500 bp long fragments were purified and amplified by a standard PCR. The mixture of mutagenized *ssgB* genes produced by error-prone PCR was then ligated as NdeI-HindIII fragments under the control of the natural *ssgB* promoter in pJPM2. The DNA was subsequently transformed into protoplasts of the *S. coelicolor ssgB* null mutant, thereby generating a collection of *Streptomyces* colonies each expressing a variant of SsgB from the natural *ssgB* promoter. For the eGFP fusions, we amplified the *ssgB-eGFP* fusion from the adapted cosmid STL2 by using primers pSsgB_fw and GFP_rv. The centre part of *ssgB* was then replaced by SacI, XhoI digestion (two naturally occurring restriction sites within *ssgB*) with the substitution version of *ssgB*. Mutations were verified by DNA sequencing.

### 5.3. Scanner-based imaging

Plates were incubated at 30°C on a flat-bed CCD scanner and imaged every 30 min for 7 days. Automated scanning was performed using Quickscan (www.burrotech.com) activated by the Windows task scheduler. Hereafter the image stack was analysed for grey values using imageJ/FIJI. This was achieved by drawing an equal sized circle in the middle of the grown colonies and measuring the grey level intensity of the stack via the Measure stack plugin of ImageJ.

### 5.4. Automated spore measurements

For each image in the folder, the scale is set to correspond to the microscope's settings, hereafter the image size is increased to optimize for averaging of pixels values at later stages of the macro. After increasing the image size by 5% in 20 consecutive operations, the edge detection filter of ImageJ is applied. Everything above the default threshold is defined as a proper edge. The holes are filled and the spores and hyphae that are detected in this manner are defined as ‘in focus’. The original file is thresholded with default settings and combined with an AND operation with the sharp spores. Particles that fall within the range of spores are analysed, with minimum size of 0.65 μm^2^, and a roundness value between 0.75 and 1.

### 5.5. Microscopy

#### 5.5.1. Live/dead staining and DNA quantification

For live/dead staining, *Streptomyces* strains were grown on SFM agar plates and after 7 days coverslips were pressed onto the colony and mounted in PBS containing 10 µM syto 9 and 10 µg ml^−1^ PI. Fluorescence and light microscopy were performed as described previously [52].

For DNA quantification, *Streptomyces* colonies were grown against coverslips (at 45° angle) on SFM agar, and after 7 days the agar samples were fixed with 2% paraformaldehyde for 5 min and washed with 70% ethanol. Subsequently spores were stained with 1 µM Syto green (ThermoFischer) and imaged with a zeiss Axioplan 2, with 470/40 excitation and 525/50 emission. For localization studies, coverslips were immediately imaged without pretreatment, using the same microscope. For DNA quantification the total intensity of each separate spore in a spore chain was measured and to circumvent staining variation the median value of each spore chain was set to 1 to normalize the data. To have enough data for normalization, we used spore chains that consisted of a minimum of 10 spores. All images were background-corrected, setting the signal outside the hyphae to 0 to obtain a sufficiently dark background. Images were processed using Adobe Photoshop CS4 and FIJI.

#### 5.5.2. Fluorescence recovery after photobleaching

FRAP was performed with a Zeiss Imager LSM 510, using 488 nm excitation and 505–550 nm detection as described [30].

#### 5.5.3. Electron microscopy

Cryo-scanning electron microscopy (cryo-SEM) was performed as described [53]. For SEM imaging of individual spores, impression prints of 7-day old confluent plates were obtained and fixed with 1.5% glutaraldehyde in PBS. After 15 min fixation, samples were dehydrated using a graded series of acetone (70%, 80%, 90%, 96%, 100%, 15 min each) and subsequently critical point dried. Before examination, 10 nm Platinum/Paladium was sputter coated on the sample to prevent charging during imaging. All images were obtained with a Jeol 7600 at 5 kV and a working distance of 8 mm.

For TEM, small cubes of colonies were fixed with 1.5% glutaraldehyde in PBS for 1 h, postfixed with osmium tetroxide (1%) for 1 h and dehydrated with a graded ethanol series (70%, 80%, 90%, 100% 15 min each). Ultrathin sections of 70 nm were cut and examined using a Jeol 1010. Purified FtsZ (15 µM) was combined with an equimolar amount of wild-type SsgB, or one of its mutants (SsgB-E120G, SsgB E120A and SsgBΔC) in a reaction buffer of 20 mM HEPES, pH 7.5, 150 mM KCl, 2.5 mM MgCl_2_. Polymerization was initiated by the addition of 2 mM GTP to the assembly reaction. 50 µl reaction mixture was incubated for 2 min at 37°C. 15 µl aliquot was placed on a carbon-coated copper grid and negatively stained with 2% uranyl acetate for 10 min, then washed and dried. Images were collected using transmission electron microscope (Jeol 1010) operated at 70 kV with 670 pA cm^−2^ density and recorded on a camera.

### 5.6. Co-pelleting assay

For pelleting experiments, purified FtsZ, with or without equimolar ratios of wild-type SsgB, or its mutants (SsgB-E120G, SsgB E120A and SsgB*Δ*C) were mixed at a final concentration of 15 µM. The samples were pre-spun at 50 k r.p.m. in a Beckman 70 Ti rotor for 30 min. Then supernatants were transferred to new tubes and 2 mM MgCl_2_ and GTP were added. After 10 min of incubation at 30°C, the tubes were centrifuged at 65 k r.p.m. at 20°C for 30 min. The supernatants were removed for analysis and pellets were washed with the same buffer aside from the proteins. Pellets were dissolved in SDS gel loading buffer. All the samples were analysed on a 4–16% Pre-cast SDS-PAGE gel (BIO-RAD).

### 5.7. Protein expression and purification

For expression of His_6_-tagged fusions of SsgB and FtsZ in *E. coli,* the entire coding region of *ssgB* or its mutants was cloned from the genomic DNA of *S. coelicolor* as NdeI-HindIII fragments and ligated into pET28a (Novagen). Sequences of *S. coelicolor ftsZ* were codon optimized prior to cloning into pET28a. SsgB was overexpressed in *E. coli* Rosetta™ 2 (DE3) pLysS strain as a 157 aa-long fusion protein containing the 137 aa native polypeptide and an N-terminal tag, GSSHHHHHHSSG. SsgB point mutants E120G and E120A, or its deletion mutant (*Δ*SsgB, 1–114 aa; lacking the C-terminal 11 aa), were expressed in the same way. FtsZ was produced as C-terminal His-tag fusion. His_6_-tagged FtsZ proteins were purified using routine methods as described [54].

### 5.8. Oligomerization studies of SsgB

The polymerization state of SsgB was analysed by size-exclusion chromatography on an ÄKTA-pure system using column Superdex™ 75 Increase 10/300 GL. The elution solvent was 20 mM HEPES, 150 mM KCl, 2.5 mM MgCl_2_, 5% Glycerol, 1 mM DTT, at pH 7.5, and the flow rate was 0.1 ml min^−1^. Absorbance at 280 nm was monitored. Conalbumin, γ-globulin, ovalbumin, myoglobin and vitamin B12 were used as molecular weight standards.

### 5.9. Crystallization and structure determination

SsgB trimer crystals were grown at 18°C by using the sitting-drop vapour-diffusion method. In a typical experiment, 1 µl of the protein stock (6.2 mg ml^−1^ protein) was mixed with 1 µl of a reservoir buffer consisting of 0.2 M sodium chloride, 0.1 M sodium/potassium phosphate (pH 6.2), 50% PEG200. Crystals were moved to the same condition supplemented with 20% glycerol and flash-frozen in liquid nitrogen. Data were collected at beam line ID30B [55] at ESRF (Grenoble, France, 2017). Images were collected with a 0.15° oscillation angle and an exposure time of 0.037 s per frame at 100 K. Crystals form diffracted to 2.1 Å for SsgB trimer. The data were processed with XDS [56] and scaled using AIMLESS [57] from CCP4 package [58]. Phases of SsgB trimer were calculated by molecular replacement with SsgB*^Tfus^* PDB entry 1C3M [36] as a model using MOLREP [59]. The structures were finalized by manual building in COOT [60] and refined with REFMAC [61]. Residues were in the most favoured regions of the Ramachandran plot [62] as determined by PROCHECK [63]. Crystallographic data are summarized in electronic supplementary material, table S6. SsgB trimer structure was deposited in the PDB with code 6SLD.

### 5.10. Molecular simulation

All atom molecular dynamics (AAMD) simulation of SsgB wild-type and SsgB-E120G were prepared and run using Gromacs 2016 package [64]. The simulated protein, wild-type or mutant, was centred in a cubic box surrounded by water molecules and counter ions Na^+^ were added to keep the total charge of the simulation box to be zero. The bonded and non-bonded parameters were obtained from AMBER99SB force field [65]. The SPCE [66] water model was used. The particle-mesh Ewald method was used to treat the long-range electrostatic interactions [67]. A cutoff of 12 Å was used for non-bonded interactions. Temperature was maintained at 300 K using v-rescale thermostat [68]; pressure was maintained at 1 bar Parrinello–Rahman barostat [69,70]. The simulation box was cubic of length around 9.2 nm, with periodic boundary conditions applied to all dimensions. All simulated systems were stabilized though energy minimization, short NVT (10 ps) and short NPT (5 ns) MD simulations to relax to favourable conformations. NPT simulations at 300 K and 1.0 bar were performed upon the stabilized structures and 50 ns trajectories were collected for our analysis.

## Data accessibility

The atomic coordinates of SsgB from *Streptomyces coelicolor* have been deposited in the Protein Data Bank (PDB) with accession code 6SLC.
